# Investigation of *Coxiella burnetii* distribution in a Scottish dairy cattle herd with history of stillbirths

**DOI:** 10.1002/vetr.5815

**Published:** 2025-12-03

**Authors:** Jo E. B. Halliday, Adri Aparicio‐Chagolla, Ryan W. Carter, Richard Vazquez, Lorenzo Viora, Colin Mason, Kathryn J. Allan

**Affiliations:** ^1^ School of Biodiversity, One Health & Veterinary Medicine University of Glasgow Glasgow UK; ^2^ Veterinary Services, Scotland's Rural College Dumfries UK

## Abstract

**Background:**

*Coxiella burnetii* is a bacterial pathogen that can cause abortion and reproductive disease in livestock. In the UK, *C. burnetii* affects many dairy cattle herds, although the infection dynamics are poorly understood. Our study was performed to investigate infection patterns within a dairy cattle herd in Scotland that had experienced stillbirths attributed to *C. burnetii* infection.

**Methods:**

Different management groups within the affected herd were sampled. Serology and qPCR testing of vaginal swabs were performed to investigate infection status. Regression analyses were performed to evaluate associations between diagnostic results and variables describing calving status and farm site.

**Results:**

*C. burnetii* infection was detected in all management groups within the herd. The highest seroprevalence was detected in pre‐calving heifers (78.6%) and the highest bacterial loads were detected in post‐calving animals.

**Limitations:**

These data represent a sample from one farm and testing for a single pathogen shedding route, which limits the generalizability of our findings.

**Conclusions:**

*C. burnetii* infection is widespread within this affected herd. Marked differences were observed between the management groups, which may be explained by variables including pregnancy stage or environmental factors. Further work is needed to understand the implications of these results for the wider UK dairy sector.

## INTRODUCTION

*Coxiella burnetii* is a globally distributed zoonotic bacterium that has a wide host range. The bacteria causes coxiellosis in livestock and Q fever in people and is thought to be endemic to most of the world.^1^ In the UK, there is evidence that *C. burnetii* is distributed throughout dairy cattle herds, although the historic lack of active surveillance for *C. burnetii* means that the prevalence and production impacts of infection are currently poorly understood.^2^ In Scotland, over 80% of unvaccinated dairy herds sampled by bulk milk screening in 2014‒2015 were seropositive for *C. burnetii*, indicating essentially ubiquitous presence of the bacteria in dairy herds.^3^ Data are limited on the within‐herd epidemiology of infection as well as the clinical, economic and public health impacts of livestock infection in the UK context.^2^, ^3^ Notably, *C. burnetii* was recently reclassified as a reportable disease in the UK in 2021, leading to increased awareness and investigation of this pathogen.^4^ An inactivated phase I vaccine is available for use (Coxevac, CEVA Santé Animale^5^), but it is not routinely recommended for inclusion in herd health plans in the UK.^6^ The lack of evidence on infection dynamics within infected herds in the UK limits the ability of clinicians and farmers to develop evidence‐based herd health plans for the control of infection.

To date, most investigation of the impacts of *C. burnetii* has focused on the influence of infection on reproduction in small ruminants. Although many livestock infections appear to be subclinical, there is evidence of association between *C. burnetii* infection and poor reproductive outcomes in cattle, sheep and goats, including sporadic abortion, premature delivery, stillbirth and weak offspring.^7^ Regardless of clinical status, infected cattle may pose an infection risk to people and other livestock species as the bacteria can be shed by multiple routes and detected in samples including vaginal mucus and birthing fluids, milk, faeces and urine.^8^ Bacterial load is considered to be a predictor of clinical significance of *C. burnetii* infection in livestock, where compatible clinical signs exist.^1^, ^9^, ^10^ The Ct value obtained from qPCR assays has a reciprocal relationship with bacterial load, where lower Ct values are associated with higher bacterial loads and vice versa, and can be used as a proxy measure for bacterial load in clinical samples.^11^ However, shedding via all routes can be intermittent and misclassification of individual infection status can occur with samples of all types.^1^, ^12^ Similarly, while serological testing is a helpful indicator of exposure at the herd level, it is an imperfect indicator of individual infection status and herd prevalence. Antibody dynamics and bacterial shedding have been described to vary in a wave‐like cycle over time in cattle populations where infection is endemic.^13^ Bacterial shedding by seronegative animals can occur^14^, ^15^, ^16^ and both seropositivity and shedding status of animals within an infected herd may vary depending on the stage of infection (e.g., acute or chronic).^13^ These factors make it challenging to understand both the epidemiology and the impacts of *C. burnetii* infection in cattle herds.

This opportunistic study was initiated following an investigation of a series of stillbirth events reported during 2021 on a single dairy cattle farm in Scotland.^17^ An increase in stillbirths in heifers, observed during the 3‐month period from February to April 2021, prompted submission of samples for stillbirth investigation at Scotland's Rural College (SRUC). From May to July 2021, five full‐term stillbirths from heifers were submitted for abortion investigation. *C. burnetii* testing was performed based on clinical suspicion due to histopathological evidence of placentitis with gram‐negative, acid‐fast (positive modified Ziehl‒Neelsen) intracellular bacteria within lesions. Immunohistochemistry and qPCR for *C. burnetii* demonstrated presence of the bacteria within placental tissue and lesions leading to a diagnosis of Q fever‐associated stillbirth in three of the five stillbirth submissions.^18^ Initial on‐farm serological investigations prompted by the stillbirth diagnoses demonstrated variable patterns of seroexposure within heifer groups kept at this farm.^17^

This investigation was performed to enhance our understanding of *C. burnetii* infection patterns within this clinically affected dairy herd. The aims of this study were to characterise the *C. burnetii* serostatus and *C. burnetii* shedding status of an opportunistically selected subset of animals from different management groups. Diagnostic findings were compared between management groups to help understand how infection status varies throughout the herd.

## MATERIALS AND METHODS

This cross‐sectional study was performed in a single dairy herd in the UK with 362 Holstein lactating dairy cows (130 primiparous and 242 multiparous) and 300 youngstock at the time of sampling. All cows were milked through an automatic milking system and housed year‐round in free‐stall sheds with deep sand cubicles. The mean farm 305‐day mature‐equivalent milk production of the herd (from the Cattle Information Service monthly milk recordings) was 10,771 kg. Replacement heifers are reared and bred at a separate unit (approximately 1 km from the main farm), and moved to the main farm 8 weeks prior to calving. The farm follows a year‐round calving pattern, with cows artificially inseminated using conventional or sexed Holstein semen from high‐fertility sires. Heifers are bred either through artificial insemination or natural service by a homebred bull. Sire and breeding method selection is determined by the farmer. All animals are exclusively managed by the holder and/or a family member, with the same equipment shared across all premises. No animals at either farm site had been vaccinated against *C. burnetii* at the time of this study.

Initially, samples used in this study were collected as part of a clinical investigation to understand the extent of *C. burnetii* infection across this herd. Subsequent enrolment in research studies, access and analysis of archived samples and data were performed under ethical approval granted by the Research Ethics Committee of the School of Veterinary Medicine, University of Glasgow (EA34/22). Written informed consent was obtained from farm representatives for access to archived samples and approval for data analyses.

Different age‐based management groups were targeted for sampling. A sample of 25 individuals from each group was targeted, which was sufficient to allow detection of *C. burnetii* in each group, assuming a prevalence of 20% or greater, test sensitivity of 90% and confidence of 95%. Animals at the heifer rearing unit and main farm were sampled in five groups: (1) pre‐bulling heifers (PBH) at the heifer rearing unit; (2) pre‐calving heifers at the heifer rearing unit (PCH‐HU); (3) pre‐calving heifers at the main farm (PCH‐MF); (4) post‐calving primiparous cows 1‒30 days in milk in the main herd (PCIM) and (5) adult cows in the main herd at any lactation stage (ADU). Incomplete data were captured for some animals in the PCIM and ADU groups, so these were combined for all analyses and reported as a single group of post‐calving animals at the main farm (ADU‐MF) (.

Sampling was conducted between February and May 2022. The mean, maximum and minimum external temperatures recorded by the UK Met Office at weather stations close to the farm between 1991 and 2020 were 13.2°C, 19.7°C and 7.2°C, respectively, with a mean yearly rainfall total of 918 mm.^19^ Blood samples and vaginal swabs were collected from animals that were selected opportunistically within each management group. Blood samples in vacutainer tubes were stored upright at 4°C until processing for serum separation within 48 hours. Vaginal swab samples were collected using viscose swabs (Technical Service Consultants), with the swab tip stored immediately in 1 mL DNA/RNA shield (Zymo Research) to inactivate infectious agents and preserve nucleic acid integrity prior to molecular testing. Swabs were stored at ‒20°C and then heat treated at 70°C for 60 minutes prior to DNA extraction.

Serum samples were stored at ‒20°C and then transferred to BioBest Diagnostics (Milton Bridge), where serological testing for exposure to *C. burnetii* was conducted using the Prio CHECK Ruminant Q Fever Ab Plate Kit (Thermo Fisher Scientific). This ELISA detects total antibodies raised against phase I and phase II antigens of *C. burnetii*. Serum samples with sample positive ratio greater than 40 were classified as positive for *C. burnetii* exposure.

DNA was extracted from swabs using the (Qiagen, Hilden, Germany) DNeasy Blood & Tissue kit. One negative extraction control was included for every 20 samples extracted. The qPCR assay for *C. burnetii* targeted the *IS1111* insertion sequence and was performed using the Qiagen RotorGene Q platform.^11^, ^20^ Reactions were carried out in 20 µL volumes comprising of 10 µL of 2× QuantiNova mastermix, 0.8 µL of 10 µM forward and reverse primers, 0.8 µL of 5 µM probe, 2.6 µL of nuclease‐free water and 5 µL of sample DNA. The sequences for forward and reverse primers were as follows—IS1111_For: 5′‐CATCACATTGCCGCGTTTAC‐3′ and IS1111_Rev: 5′‐GGTTGGTCCCTCGACAACAT‐3′. The probe sequence used was IS1111_Probe: 5′‐FAM‐AATCCCCAACAACACCTCCTTATTCCCAC‐BHQ1‐3′. Cycling conditions comprised an initial heat‐activation step of 95°C for 2 minutes, followed by 45 cycles of 95°C for 5 seconds and 60°C for 5 seconds. The samples were tested in single reactions and all runs included negative controls (extraction controls and no template controls) and positive controls. A qPCR run was considered valid when all negative controls showed no amplification and the positive controls were amplified with Ct 40 or less.

Swab sample status was classified according to predicted clinical significance of the *C. burnetii* load detected based on the Ct values obtained by qPCR. The classifications applied were based on published literature and previous work by this group (K.A., J.H. and R.C.). Samples with no evidence of qPCR amplification using a Ct cut‐off of 40 or less were classified as negative; samples with Ct values 40 or less but greater than 27 were classified as positive for DNA detection and samples with Ct 27 or less were classified as high‐load positive.^1^, ^9^, ^18^, ^21^

Regression analyses were performed in R (version 4.2.1^22^) to evaluate evidence of associations between explanatory variables and diagnostic outcome variables. Logistic regression models were used to evaluate evidence of associations between explanatory variables and the two diagnostic outcomes of serostatus (ELISA positive vs. negative) and qPCR status (where positive and highly positive animals were combined into a single category and compared to negative animals). The explanatory variables considered were farm site (main farm or heifer unit) and animal calving status (pre‐ or post‐calving). No post‐calving animals were sampled (or existed) at the heifer unit so these variables were not modelled together and only univariable assessments were performed. In addition, linear regression models were used to investigate the associations between calving status (pre‐ and post‐calving) and observed Ct values obtained from the IS111 qPCR assay, which were used as a proxy for bacterial loads shed in vaginal secretions.

## RESULTS

**FIGURE 1:**
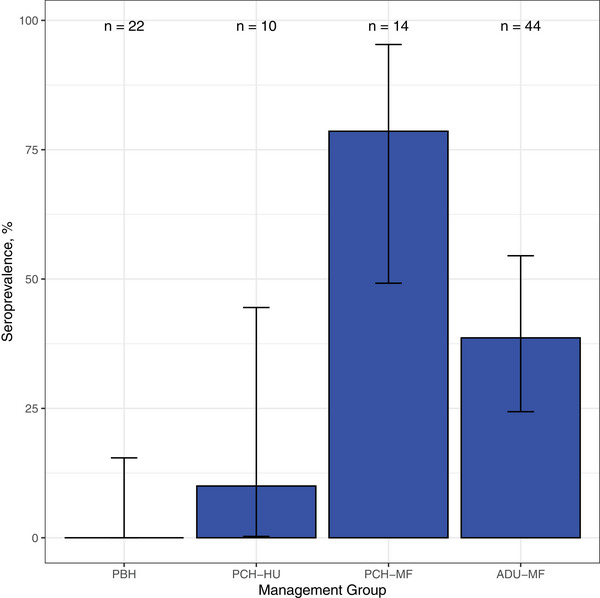
Seroprevalence of *Coxiella burnetii* exposure in different cattle management groups sampled at a single Scottish dairy farm in 2022. 95% confidence intervals are displayed for each bar. Number of observations per group is labelled above each bar. ADU‐MF, post‐calving cows (all parities) at the main farm; PBH, pre‐bulling heifers; PCH‐HU, pre‐calving heifers at the heifer rearing unit; PCH‐MF, pre‐calving heifers at the main farm.

Serum samples from a total of 90 animals were available for analysis. In total, 29 of 90 (32.2%; 95% confidence interval [95% CI]: 22.8%–42.9%) animals tested seropositive for *C. burnetii* exposure by ELISA. The numbers of animals tested and positive by ELISA in each management group are shown in Table 1. The seroprevalence by management group is shown in Figure 1. There was a significant association between serostatus and farm site, with higher odds of positive ELISA status in animals sampled at the main farm as compared to the heifer rearing unit (odds ratio [OR] 28.9, 95% CI: 5.6‒532.0, *p* < 0.001). No significant association was observed between animal serostatus and calving status.

**TABLE 1:** Composition, location and summary of *Coxiella burnetii* serological and qPCR results for each of the cattle management groups sampled at a single Scottish dairy farm in 2022.

Management group	Site	Serology results, *n*/*N* (%) [CI]	qPCR results (Ct ≤ 40), *n*/*N* (%) [CI]	qPCR results by Ct value classification, *n*/*N* (%) [CI]^a^
Pre‐bulling heifers	Heifer rearing unit	0/22 (0.0%) [0.0%‒15.4%]	5/24 (20.8%) [7.13%‒42.2%]	Negative: 19/24 (79.2%) [57.8%‒92.9%] Positive: 5/24 (20.8%) [7.1%‒42.2%] High‐load positive: 0/24 (0.0%) [0.0%‒14.2%]
Pre‐calving heifers	Heifer rearing unit	1/10 (10.0%) [0.25%‒44.5%]	9/11 (81.8%) [48.2%‒97.7%]	Negative: 2/11 (18.2%) [2.3%‒51.8%] Positive: 9/11 (81.8%) [48.2%‒97.7%] High‐load positive: 0/11 (0.0%) [0.0%‒28.5%]
Pre‐calving heifers	Main farm	11/14 (78.6%) [49.2%‒95.3%]	6/13 (46.2%) [19.2%‒74.9%]	Negative: 7/13 (53.8%) [25.1%‒80.8%] Positive: 6/13 (46.2%) [19.2%‒74.9%] High‐load positive: 0/13 (0.0%) [0.0%‒24.7%]
Post‐calving cows	Main farm	17/44 (38.6%) [24.4%‒54.5%]	47/48 (97.9%) [88.9%‒99.9%]	Negative: 1/48 (2.1%) [0.0%‒11.1%] Positive: 15/48 (31.3%) [18.7%‒46.3%] High‐load positive: 32/48 (66.7%) [51.6%‒79.6%]
Total	Both sites	29/90 (32.2%) [22.8%‒42.9%]	67/97 (69.1%) [59.6%‒78.7%]	Negative: 29/96 (30.2%) [21.2%‒40.4%] Positive: 35/96 (36.5%) [26.9%‒46.9%] High‐load positive: 32/96 (33.3%) [24.0%‒43.7%]

Abbreviation: CI, confidence interval.

^a^ Ct value result groupings: positive = Ct values ≤40 but >27; high‐load positive = Ct values ≤27; negative = no evidence of amplification with Ct values ≤40.

**FIGURE 2:**
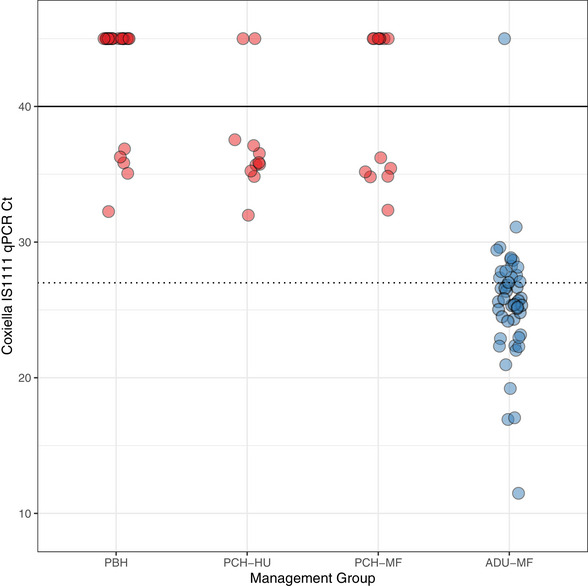
Scatterplot of qPCR Ct results obtained for *Coxiella burnetii* detection in cattle management groups sampled at a single Scottish dairy farm in 2022. Point colour indicates calving status where red denotes pre‐calving and blue denotes post‐calving. The horizontal lines indicate the thresholds applied for result classifications for positive samples with Ct values 40 or less (solid line), and samples classed as high‐load positives with Ct values 27 or less (dotted line). Points plotted at Ct value of 45 are negative samples with no evidence of amplification after 40 qPCR cycles and are included for illustration only. ADU‐MF, post‐calving cows (all parities) at the main farm; PBH, pre‐bulling heifers; PCH‐HU, pre‐calving heifers at the heifer rearing unit; PCH‐MF, pre‐calving heifers at the main farm.

Evidence of *C. burnetii* DNA was detected by IS1111 qPCR, with a Ct 40 or less in 67 of 96 (69.8%; 95% CI: 59.6%–78.7%) swabs tested (positive or high‐load positive classification). Statistically significant associations with qPCR status were observed for both farm site and animal calving status. Animals sampled at the main farm were more likely to be qPCR positive than animals sampled at the heifer rearing unit (OR 9.9, 95% CI: 3.8‒28.6, *p* < 0.001). Animals sampled post‐calving were more likely to be qPCR positive than animals sampled pre‐calving (OR 65.8, 95% CI: 12.7‒1213.4, *p* < 0.001). In qPCR‐positive animals, a range of Ct values was detected (Ct 11.5–37.6) (Figure 2). Lower Ct values, indicating higher concentrations of bacteria, were seen in the post‐calving animals as compared to pre‐calving animals. In the combined ADU‐MF population, 32 of 48 (66.7%; 95% CI: 51.6%–79.6%) animals were classified as high‐load positives compared to other management groups (all heifers), in which no high‐load positives were detected. Comparison of Ct values between pre‐ and post‐calving animals showed that post‐calving animals had significantly lower Ct values than pre‐calving animals (estimated Ct difference of ‒10.2, 95% CI: ‒11.9 to ‒8.52, *p* < 0.001), indicating a significantly higher bacterial load in post‐calving animals.

## DISCUSSION

This investigation reveals evidence of *C. burnetii* infection throughout a dairy cattle herd with a history of stillbirths attributed to *C. burnetii*. Evidence of serological exposure to *C. burnetii* was observed in all management groups except for PBH. Animals sampled on the main farm were significantly more likely to be seropositive compared to animals sampled at the heifer rearing unit. There was no association between serostatus and calving status. Animals at the main farm were also significantly more likely to be shedding *C. burnetii* in vaginal fluids (as indicated by a qPCR‐positive result), but this site association may be confounded by animal calving status, as calving status was also a significant predictor of bacterial shedding. Significantly lower Ct values, indicative of a higher, clinically significant, bacterial load, were observed in post‐calving animals than in pre‐calving animals, suggesting that post‐calving animals pose the highest risk of onward transmission of infection to other cattle and to people.

The pattern of serological exposure observed in this herd suggests that most pre‐calving heifers seroconvert around the time that they transition from the heifer rearing unit to the main farm site (Figure 1). This pattern could be explained by environmental or biological factors. Aerosol inhalation and environmental contamination are major routes of *C. burnetii* transmission between and within herds^1^, ^23^ so higher seroexposure seen in animals sampled at the main farm may be explained by an increase in infection pressure when heifers move to the main farm and enter an environment contaminated with *C. burnetii*. The differences in exposure to *C. burnetii* seen between animals at the two sites (Figure 1) may be due to differences in environmental conditions that influence pathogen presence and persistence. The two sites are relatively close to each other though and well connected in terms of animal movements between sites. On this farm, primiparous heifers often share calving pens with multiparous cows, allowing ample opportunity for pathogen transmission. However, the detection of one seropositive animal and multiple qPCR‐positive individuals in the pre‐bulling and pre‐calving animals at the heifer rearing unit provides evidence that some animals are exposed to *C. burnetii* prior to arrival at the main farm (Table 1 and Figure 2). This therefore suggests that other biological factors, such as the stage of reproductive cycle and method of breeding used, may be driving the serological and shedding patterns observed here. Previous work in Spanish dairy herds has demonstrated that seropositivity is greater in first or second terms of pregnancy compared to non‐pregnant cows, and that seroexposure is more likely to be observed in primiparous as compared to multiparous cows.^24^ The lower seroprevalence seen in the post‐calving animals (ADU‐MH) as compared to PCH‐MF (38.6% vs. 78.6%, Figure 1) is also consistent with previous studies that have demonstrated that individual‐level antibody titres decline during the postpartum period and/or in multiparous cattle.^14^, ^24^ Seroconversion during pregnancy may also result from immunosuppression in the last third of gestation that allows for recrudescence of existing *C. burnetii* infection in previously infected animals.^8^ Other studies have demonstrated a cyclical variation in seroconversion within chronically infected herds as well as more nuanced patterns when antibodies associated with the different phases of *Coxiella* infection (i.e., phase I vs. phase II) are investigated.^13^, ^25^ Further investigation would be needed to disentangle the various influences of environment, parity, pregnancy stage and the stage and state of the herd infection (e.g., an acute recent infection or a chronic endemic infection) to fully understand the implications of the serological results seen in this population.

High levels of *C. burnetii* shedding, as defined by qPCR detection of *C. burnetii* DNA, were also detected across farm sites and management groups, but bacterial loads were significantly higher in post‐calving animals when compared to pre‐calving animals as indicated by the Ct values obtained from vaginal swab samples (Figure 2). The only animals that met our ‘highly positive’ classification, designed to indicate likely associations with clinical significance, were in the adult (post‐calving) group sampled at the main farm (ADU‐MF) (Table 1). Pregnancy stage has been shown to influence the prevalence and load of *C. burnetii* shedding in infected cattle, with the highest prevalence of shedding occurring immediately postpartum, with recovery to a non‐shedding state by 90 days postpartum.^8^ The mechanism for this is not fully understand but factors such as reduced immunity, increased physiological stress around parturition, and the mechanical effects of uterine and cervical involution following parturition may all contribute to this pattern.^26^, ^27^, ^28^ It is also worthwhile to note that bacterial load detected in clinical samples from abortion and stillbirth cases in the immediate postpartum period (i.e., 0‒7 days postpartum) has also been used to differentiate between ruminant abortion cases caused by *C. burnetii* versus incidental findings in abortions caused by other pathogens.^1^, ^9^, ^10^, ^18^, ^21^ Therefore, while shedding is a normal feature in the postpartum period in chronically infected animals, the magnitude of bacterial shedding may provide important clinical information regarding the pathological impact of infection in these animals. Although the animals sampled in this study were selected opportunistically and were not reported to have experienced an abortion or stillbirth, we know that *C. burnetii* abortions have occurred previously on this farm,^17^, ^18^ and the very high bacterial loads observed in some animals in our study (e.g., 32 of 48 ADU‐MF animals sampled within the main herd) indicate the likely clinical importance of ongoing infection within this herd. Furthermore, the shedding of high loads of bacteria has important zoonotic implications for farmers and farm staff working in an environment with high potential for aerosolised *C. burnetii* bacteria.

This study has some limitations. This study was conducted on a single farm and the small sample sizes in some management groups limit the precision of some of our findings. Sampling using a single shedding route may have reduced the sensitivity of detection of our approach.^12^ The ELISA test used detects antibodies against both phase I and phase II antigens of *C. burnetii* and we are therefore unable to differentiate acute and chronic infections. However, the ELISA test that we used is one which is commercially available and is recommended for diagnosis of *C. burnetii* infection within livestock herds in the UK. The data presented here are representative of ‘real‐world’ test results and data available for UK‐based farmers and veterinarians on which to base clinical decisions and therefore have value in providing context for the interpretation of clinical results generated as part of everyday herd health investigations. Due to incomplete data capture, we cannot investigate the influence of parity or exact timing of parturition relative to sampling on the diagnostic findings from this study. No data were captured on milk yield to assess the influence of *C. burnetii* infection on yield. As a result of these limitations, it is not possible to link individual animal infection data to individual‐level production and reproductive performance in this herd. However, the history of clinical disease associated with *C. burnetii* within this herd provides evidence of its clinical impact at herd level. The high seroprevalence and shedding rates seen in this herd are in accordance with several studies reported in the wider European literature, including studies from high‐producing herds,^14^, ^29^ suggesting that the general patterns seen in our results may indeed be replicated in other herds.

Understanding infection dynamics within dairy herds has important implications for designing intervention strategies to control *C. burnetii*. Vaccination is considered one of the mainstays of control of *C. burnetii* in livestock in Europe,^30^ although research studies have shown varying levels of association between vaccination and improved reproductive disease metrics in dairy cattle.^31^, ^32^ Vaccination is also an option for infected dairy cattle herds in the UK, but our study highlights some practical challenges to delivering an effective vaccine intervention. For example, the vaccine datasheet for Coxevac recommends vaccination of non‐infected, non‐pregnant animals to reduce the risk of *C. burnetii* shedding.^5^ In our study, although heifers were unlikely to test seropositive to *C. burnetii* (Figure 1), shedding was still detected in vaginal fluids of around 20% of PBH (Figure 2). If other herds prove similar to our study herd, early vaccination of replacement stock may well be needed to meet datasheet recommendations of initial vaccination prior to first exposure. Furthermore, the probability of shedding has been shown to be reduced, but not necessarily eliminated, in vaccinated animals,^5^, ^30^ so public health measures may still be needed for farm staff working closely with infected animals and birth products. Finally, there are currently limited data regarding the production and economic benefits of *C. burnetii* vaccination in dairy cattle in the UK,^30^ so further investigation into the clinical impact of infection would be valuable to help understand the true cost of this often‐overlooked infection in the UK dairy cattle sector.

## AUTHOR CONTRIBUTIONS

*Conceptualisation*: Jo E.B. Halliday, Richard Vazquez, Lorenzo Viora, Colin Mason and Kathryn J. Allan. *Data curation*: Jo E.B. Halliday. *Formal analysis*: Jo E.B. Halliday, Adri Aparicio‐Chagolla and Kathryn J. Allan. *Funding acquisition*: Jo E.B. Halliday, Adri Aparicio‐Chagolla, Richard Vazquez, Lorenzo Viora, Colin Mason and Kathryn J. Allan. *Investigation*: Jo E.B. Halliday, Adri Aparicio‐Chagolla, Ryan W. Carter, Richard Vazquez, Lorenzo Viora, Colin Mason and Kathryn J. Allan. *Methodology*: Jo E.B. Halliday, Richard Vazquez, Lorenzo Viora and Kathryn J. Allan. *Project administration*: Jo E.B. Halliday, Richard Vazquez, Lorenzo Viora and Kathryn J. Allan. *Resources*: Colin Mason. *Supervision*: Jo E.B. Halliday, Richard Vazquez, Lorenzo Viora and Kathryn J. Allan. *Visualisation*: Jo E.B. Halliday and Kathryn J. Allan. *Writing—original draft*: Jo E.B. Halliday, Adri Aparicio‐Chagolla and Kathryn J. Allan. *Writing—review and editing*: Jo E.B. Halliday, Adri Aparicio‐Chagolla, Ryan W. Carter, Richard Vazquez, Lorenzo Viora, Colin Mason and Kathryn J. Allan.

## CONFLICT OF INTEREST STATEMENT

Commercial testing at SRUC of two bulk milk tank samples for *C. burnetii* by qPCR and a subset of serology samples (*n* = 24) from this farm were paid for by CEVA Animal Health as part of the initial clinical investigations. R.V. and L.V. also received funding from CEVA Animal Health for previous studies on cattle reproduction.

## ETHICS STATEMENT

Enrolment in research studies, access and analysis of archived clinical samples and data were performed under ethical approval granted by the Research Ethics Committee of the School of Veterinary Medicine, University of Glasgow (EA34/22).

## Data Availability

The data that support the findings of this study are available upon request from the corresponding author. The data are not publicly available due to privacy or ethical restrictions.
